# The Effect of Different Obturation Techniques in Primary Teeth on the Apical Microleakage using Endoflas: A Comparative In Vitro Study

**DOI:** 10.1155/2023/4982980

**Published:** 2023-03-31

**Authors:** Alaa Fadhil Irzooqee, Aseel Haidar M. J. Al Haidar, Maha Abdul-Kareem

**Affiliations:** ^1^Department of Pediatric and Preventive Dentistry, College of Dentistry, University of Baghdad, Baghdad, Iraq; ^2^Department of Pediatric and Preventive Dentistry, College of Dentistry, Al-Mustansiriya University, Baghdad, Iraq

## Abstract

**Objectives:**

This study was carried out to quantitatively evaluate and compare the sealing ability of Endoflas by using different obturation techniques.

**Materials and Methods:**

After 42 extracted primary maxillary incisors and canines were decoronated, their canals were instrumented with K files of size ranging from #15 to #50. In accordance with the obturation technique, the samples were divided into three experimental groups, namely, group I: endodontic pressure syringe, group II: modified disposable syringe, and group III: reamer technique, and two control groups. Dye extraction method was used for leakage evaluation. Data were analyzed using one-way ANOVA and Dunnett's T3 post hoc tests. The level of significance was set at *p* < 0.05.

**Results:**

Endodontic pressure syringe had significantly less leakage than the modified disposable syringe and reamer techniques (*p* < 0.05). Meanwhile, no significant difference was found in the mean leakage between the modified disposable syringe and the reamer techniques.

**Conclusion:**

Amongst all the techniques used in this study, endodontic pressure syringe could be preferred as an obturation technique in primary teeth when used with Endoflas obturation material because of its potential to provide good apical seal.

## 1. Introduction

Maintaining each primary tooth as a fully functional component of the dental arch is the primary goal of pulp therapy for primary dentition. This maintenance promotes appropriate occlusion, mastication, phonation, and swallowing, as well as the maintenance of the space needed for the emergence of the permanent successor teeth and to get rid of any negative psychological effect that could be induced by tooth loss [1, 2]. In primary teeth, pulpectomy is the favorable treatment for the presence of irreversible inflammation or necrosis of the pulpal tissue that needs to be extirpated and properly removed so that the root canals could be obturated with appropriate filling material [3].

The primary goal of pulpectomy is to fill the endodontic space in three dimensions to create a fluid-tight barrier that is stable over time and to protect the periradicular tissues from the leakage of the oral micro-organisms and their by-products to prevent root canal infection [4]. Obturation with minimum voids and to an optimum length is necessary for successful pulpectomy [5]. It requisites the use of a resorbable material that is similar to that of physiological primary root resorption to produce an airtight seal with antimicrobial qualities, inhibiting the growth of resident bacteria and stimulating periapical healing [6].

Apical microleakage, which is the entrance of oral fluids along the interface between a tooth structure and the obturation materials, is the main cause of endodontic failure [7–9]. It is influenced by the presence or absence of smear layer, the physical and chemical properties of root-canal-filling materials, and the technique used [10]. These influences confirm the necessity to use a technique and a material that could produce a good hermetic seal with minimum voids [11]. Therefore, the sealing ability of the root-canal-filling material in primary teeth is affected mostly by the material's capacity to adhere to the walls of the root canal and the method used to deliver this material into the root canal [12].

Numerous materials, including zinc oxide eugenol, iodoform paste, and calcium hydroxide, have been evaluated for their efficacy as root-canal-filling materials. However, for primary teeth, none of them has shown all the characteristics necessary for an ideal root-canal-filling material, especially the critical property of having a rate of resorption that corresponds to the physiologic root resorption of the primary teeth [13]. Zinc oxide eugenol is resistant to the resorption that may eventually aid in a deflected successor. Furthermore, its antimicrobial efficacy is limited. Calcium hydroxide resorbs faster than the normal root resorption of primary teeth; it creates a “hollow tube” effect, in which the root canal remains unfilled and becomes infected due to the saturation of the canal with tissue fluid [14, 15].

Endoflas (Sanlor, Colombia), produced in South America, is composed of three materials: ZOE, Ca(OH)_2_, and iodoform. Compensating the drawbacks of one component with the benefits of others is the rationale behind this combination. The majority of studies found that Endoflas resorption was comparable to the physiological root resorption without producing the hollow tube effect which is a necessary component of the optimum obturating material for primary teeth [14, 16, 17]. Endoflas exceeds other obturation materials in primary teeth in terms of reducing interradicular radiolucency. Due to the presence of Ca(OH)_2_ and iodoform, this reduction could be linked to its remarkable healing properties and broad antibacterial activity [16]. Moreover, the extruded material resorbs without intracanal resorption, leading to maintaining an airtight seal [17]. As a result of its antimicrobial qualities, Endoflas can aid in regression of furcal radiolucency and total bone regeneration, thus it becomes superior to ZOE and Metapex. Due to its hydrophilic properties, Endoflas could also be recommended as an obturating material even in mild, humid canals [18]. According to the results of recent investigations, Endoflas could be used as a good obturating material because it contains antibacterial properties that could sterilize hard-to-reach accessory canals and dentinal tubules [19, 20].

In pediatric endodontic literature, several obturation techniques have been developed to optimize the outcome of root canal treatment, such as endodontic pressure syringes, modified disposable syringes, jiffy tubes, insulin syringes, tuberculin syringes, reamer, plugger, hand-held lentulospiral, and motor-driven lentulospiral [21].

Apical microleakage of root-canal-filling techniques was evaluated using dye penetration, fluid filtration, bacterial penetration, radioisotope, dye extraction, and electrochemical means. The dye extraction method involves immersing samples in dye and then into acid to remove all of the dye out of the interface. The optical density of the solution is measured with a spectrophotometer. Therefore, quantifying the amount of dye that leaks through the filling's margins is possible [22]. Since a hermetic seal of the root canal system in necrotic primary teeth is a substantial contributor to the success of pulpectomy treatment, the sealing ability of various obturation techniques needs to be investigated [23]. Studies have shown the sealing ability of different obturation techniques used in primary teeth and revealed that it has a high success rate when used with most commonly used obturation materials [24, 25].

Hence, this study was conducted to evaluate and compare the Endoflas sealing ability using different obturation techniques.

## 2. Materials and Method

### 2.1. Study Design and Sample Collection

An in vitro experimental study was designed using 42 extracted primary maxillary central incisor and canine teeth. The study was conducted at the Department of Pediatric and Preventive Dentistry, College of Dentistry, University of Baghdad, after receiving ethical approval from the University of Baghdad's Ethical Committee (Ref. 570 on April 17, 2022) in compliance with the Helsinki Declaration and its guiding principles [26]. The samples were selected out of 245 extracted teeth, which took about 5 months to collect. The teeth had been extracted due to various reasons, including (i) abscess, (ii) pulpitis, (iii) trauma, and (iv) orthodontic consideration. Before sample collection, the aims of the study were explained to the parents, and their permission to use their children's teeth was acquired via a consent form.

### 2.2. Teeth Selection Criteria

After careful radiographic and microscopic examination, only 42 teeth from the entire selected samples met the following inclusion criteria, as reported by Bawazir and Salama [12], with some modifications:

1. Straight roots.
2. At least two-thirds of the root is intact.
3. A length range of 15–22 mm measured from the incisal edge to the root apex.
4. An apical opening not wider than a #30 K-file (DENTSPLY Maillefer, Switzerland).

Teeth that were found to have aberrant root fracture, calcification, canal obliteration, and internal or external resorption on periapical radiographs were excluded from the study. A 2.5% NaOCl solution was used to eliminate the organic material. The selected teeth were immersed in it for 48 hr. Tissue remnants were then brushed and washed under running water and preserved in sterile distilled water until the beginning of the study [12]. The sample size was calculated statistically using G power 3.1.9.7 (program written by Franz-Faul, Universitatit Kiel, Germany).

### 2.3. Preparation of the Sample

Decoronation was done to the selected teeth, at the cementoenamel junction at 10 mm length, with a diamond disc bur and a straight handpiece [27, 28]. All the teeth were embedded in blocks of heavy body and light body silicone impression material to facilitate handling of the sample during instrumentation and obturation procedure and allow maximum stimulation of the practiced clinical condition in typical endodontic treatment of the bony socket and periodontal ligament [29]. All the teeth received standardized instrumentation procedures. A barbed broach was used to remove any pulp tissue remnants in the canals. Following the determination of the working length from the radiograph, the canals were prepared using K-files sized between #15 and #50 [30]. A 27-gauge needle 2 mm shorter than the working length was used to irrigate with 1 ml of 2.5% NaOCl between the instruments used during canal preparation to flush out debris. Following the instrumentation, irrigation to the canals was done with 2 ml of 2.5% NaOCl. Finally, administration of 1 ml of EDTA was made for 1 min, followed by irrigation with 3 ml of 2.5% NaOCl and 5 ml of distilled water. The canals were then dried with a paper point until a dry point of paper came out [31].

### 2.4. Design of the Experimental Groups

An independent person used simple randomization to divide the sample into three experimental groups (*n* = 12 for each). Each group has a positive control sample (teeth were instrumented but not obturated or varnished) and a negative control sample (teeth were obturated and completely coated with nail varnish) [27, 32].

For the experimental groups, the same operator carried out the obturation procedure in all of the groups, where Endoflas was used to obturate each root canal using the specific obturating technique assigned to it. For each technique, a standardized mixture of Endoflas was created, taking into account the technique's restrictions and the manufacturer's advice. The variation in the consistencies of the Endoflas mixture of each technique was due to the physical limitations of each technique [33]. These groups were as follows.

#### 2.4.1. Endodontic Pressure Syringe (Group I)

In accordance with the manufacturer's instructions, one scoop of Endoflas powder was mixed with one drop of liquid to obtain a thick, homogenous mixture. The mixture was loaded into the syringe's hub with a gauge of 25 needle (Pulpdent, Root Canal Pressure Syringe). As the pressure syringe had a mechanical nature (it works via a screw mechanism), the mixture was expressed through the needle. The needle was inserted 1 mm away from the apex and progressively withdrawn with each quarter turn of the screw at 3 mm intervals until the canal's orifice was filled [33].

#### 2.4.2. Modified Disposable Syringe (Group II)

**Figure 1:**
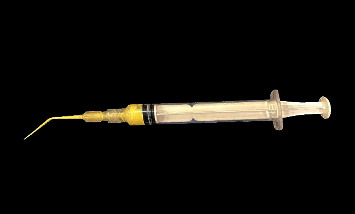
Modified disposable syringe.

Modification to the disposable syringe (Dispo Van, India) was done by adding a disposable tip to it (Meta Biomed) (Figure 1). According to Nagarathna et al. [23], one scoop of Endoflas powder was mixed with three drops of liquid to produce a thin, flowable mixture. About 1 ml of the mixture was loaded into the syringe, repeatedly tapping on a firm surface to release any trapped air bubbles. The disposable tip was fixed and the material flow was checked. At the predetermined working length, a rubber stop was placed. Insertion of the tip into the prepared canals was done until it encountered wall resistance. Then, the gradual withdrawal of the tip was done as the material was pushed [23].

#### 2.4.3. Reamer Technique (Group III)

The canals in this group were obturated using a size 30 endodontic reamer. Two scoops of Endoflas powder were mixed with one drop of liquid to create a medium consistency [6]. After the rubber stopper was placed at the desired working length, the reamer was then coated with Endoflas and introduced into the canal five to seven times for each canal with a vibratory motion and clockwise rotation until the canal's orifice appeared filled with paste [21].

**Figure 2:**
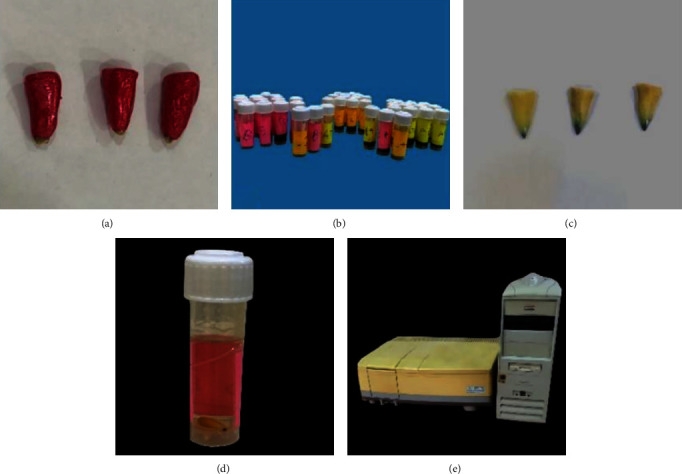
(a) After coating the sample with nail varnish; (b) the sample in methylene blue solution; (c) after washing the sample from the dye and removing the nail varnish; (d) sample in 65% nitric acid; (e) spectrophotometer device.

In all the experimental groups, the obturated samples were kept in an incubator for 24 hr at 37°C and 100% humidity to allow the setting of the Endoflas. Afterward, two layers of nail varnish were applied to the roots, except the 1 mm apical, which was left uncoated. For 24 hr, the samples were immersed in a 2% methylene blue solution. The teeth were removed and washed with tap water for 30 min. Using a surgical blade and a polishing disk, the varnish was removed. Afterward, the samples were kept in a container with 4 ml of 65% nitric acid for 3 days. After this solution was centrifuged at 4,000 rpm for 7 min, 2 ml of each sample's supernatant layer was transferred to plastic cuvettes. On an automated spectrophotometer, the optical density of the solution was measured at 550 nm by using concentrated nitric acid as a blank (Shimadzu Europe, UV-1650PC, Germany) (Figure 2) [31, 34]. An experienced examiner who was blind to the groups of the study conducted the spectrophotometer readings.

### 2.5. Statistical Analysis

Data were entered at a digital database structure on MS Excel with SPSS (version 22, Chicago, Illinois, USA). The data were analyzed using one-way ANOVA and Dunnett's T3 post hoc test (IBM Corp., Armonk, NY, USA). The significance level was set at *p* > 0.05.

## 3. Results

**Figure 3:**
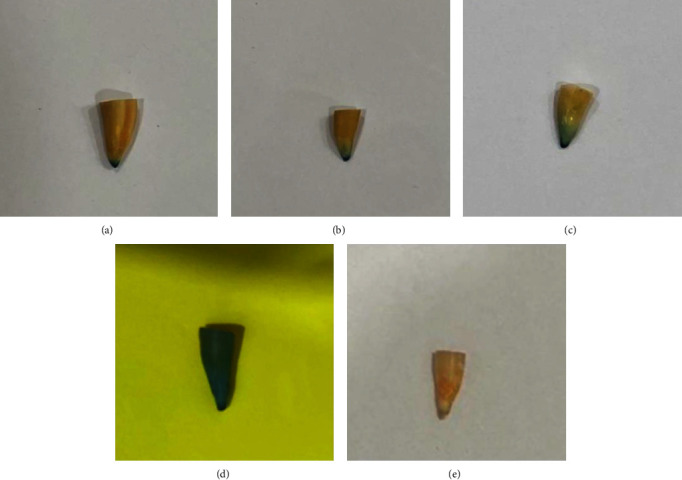
Dye leakage: (a) group I: endodontic pressure syringe; (b) group II: modified disposable syringe; (c) group III: reamer; (d) positive control; (e) negative control.

Dye leakage was detected in all three experimental groups. The positive control group demonstrated dye leakage along the entire canal length, whereas no dye leakage was found in the negative control group (Figure 3).

**Figure 4:**
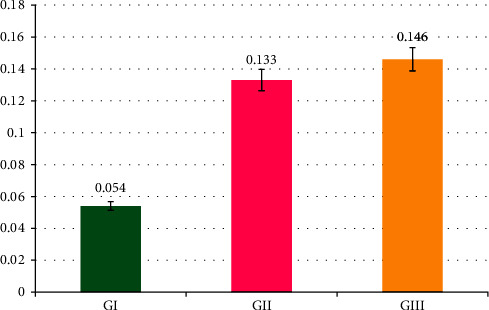
Bar graph showing the mean dye leakage value across groups.

Statistical analysis demonstrated that Group I had the lowest mean leakage value, followed by Group II, and Group III had the greatest mean leakage value (0.054, 0.133, and 0.146, respectively), as shown in Table 1 and Figure 4.

The one-way ANOVA test results showed statistical significant differences (*p* < 0.05) between the experimental groups. Dunnett's T3 post hoc test was conducted to assess the difference between each group. A significant difference was found between Groups I and II and between Groups I and III. However, the difference between Groups 2 and 3 was not significant, as shown in Table 2.

## 4. Discussion

The ability to remove the infectious micro-organisms and create good sealing by the filling material is crucial for efficient root canal treatment [35–39]. Unlike obturation in permanent teeth, performing hermitic seal root canal filling in primary teeth is challenging because primary teeth require using a resorbable and noncondensable filling material. Consequently, in the primary teeth, determination of the sealing ability of the material used to fill a root canal is mostly affected by the adherence ability of the material to the root canal walls and the technique employed to get this material into the root canal [12]. It also relies on how well the irrigation solution removes the smear layer from the root canal system [40].

This study showed that using upper primary incisors and canines is preferable because their root canals have simple and uniform morphology in contrast to the atypical differences and morphological abnormalities found in primary molars, which may negatively affect the standardization and evaluation procedures of the study [40, 41]. In this study, the apex of each selected tooth was examined microscopically to ensure the central position of the apical foramen and that it was not affected by root resorption because the resorption may result in major variations in the foramen's position, thereby affecting the study's outcome. The size of the apices selected was less than the size #30 K files to reduce variability and obtain standardization [12].

Many studies have identified an association between the presence of the smear layer and the apical microleakage. The penetration of root-canal-filling materials to the dentinal tubules is limited in the presence of this layer and there is an adaptation deficiency between the canal wall and the filling material [36, 42]. Accordingly, removing the smear layer to obtain better clinical outcomes is recommended when performing root canal therapy on primary teeth. Therefore, in this study, 17% EDTA was used to remove the smear layer [43].

In this study, methylene blue was used as the dye to assess the apical microleakage because of its characteristics, such as easy handling, high degree of staining, and low cost [44]. Methylene blue has a lower molecular weight than bacterial toxins, which may not stimulate the clinical situation [45]. The dye penetration method is the most popular method for assessing sealing ability; however, it has significant drawbacks. This method depends on randomly cutting the root into two sections without identifying which segment undergoes the deepest dye penetration, hence underestimating the dye penetration and producing unpredictable results. Moreover, leakage measurement is qualitative [4]. Contrarily, the dye extraction technique used in this study recovers all of the dye that leaked through the apex by dissolving it in acid, thus avoiding the drawbacks of sectioning the root. It also quantitatively measures the optical density of the solution by using a spectrophotometer, thus producing accurate results for the microleakage studies [46, 47].

The lowest microleakage was reported for the endodontic pressure syringe group, with significant difference with that of the modified disposable syringe and the reamer technique. The reason may be due to the design of the pressure syringe; its flexibility and thin metal tip allows for a better reach into the narrow canals toward the apex, injecting the paste uniformly and continuously, leading to a more dense and compact filling [48]. This result was in agreement with that of Monali et al. [49], who showed that the endodontic pressure syringe was the best in producing homogeneously filled canals, with fewer voids than the reamer technique, but the difference was not statistically significant.

However, the results of this study disagreed with the result reported by Kukreja et al. [50], who compared the apical seal of the endodontic pressure syringe and the incremental technique by using a radiograph through measuring the distances from the apical end of the filling material (ZOE) to the radiographic apex. According to the authors' findings, no statistically significant differences were found between these techniques. The difference between the findings of this study and those of Kukreja et al. [50] may have resulted from the difference in (i) the material used for the obturation, (ii) the technique used for the obturation where Kukreja et al. [50] used the incremental technique by endodontic plugger, and (iii) the evaluation method used.

Meanwhile, the reamer technique was found to have the highest microleakage, which differed significantly from the endodontic pressure syringe. This finding could be attributed to the structure of the hand instrument combined with the repeated removal and reinsertion during the filling up of the canals, leading to the incorporation of voids as small irregularities at the apical portion that resulted in apical microleakage [49].

This study found no significant difference in the apical microleakage between the modified syringe and reamer groups (*p* = 0.966). Although the mean of microleakage was less in the former, the air entering the barrel during material loading may cause voids in obturation with the modified disposable syringe, leading to this microleakage [23].

Using the modified disposable syringe had various advantages due to the tip's transparency. The operator could inspect the material's flow and no fracture risk could occur. Moreover, the disposable tip could be cut to the required lengths. As these tips and syringes are single-use only and could be safely disposed of, cross-contamination is not a concern [23].

No comparable studies on the apical microleakage of obturation techniques in primary teeth were identified during the preparation phase of this study. However, this study hypothesized that endodontic pressure syringe could ultimately improve the success rate of root canal treatment using Endoflas in primary teeth under routine clinical situations.

## 5. Conclusion

Amongst all the techniques used in this study, endodontic pressure syringe could be preferred as an obturation technique in primary teeth when used with Endoflas obturation material because of its potential to provide a good apical seal.

### 5.1. Limitations of the Study

A notable detail is that this study considered only one type of root-canal-filling material. As several new obturation materials are hard to obtain in Iraq, Endoflas was used in this study because of its easy accessibility and its common use in the authors' department as applicable under routine clinical situation. Therefore, further studies are required for the evaluation and comparison of more than one root-canal-filling material by using different techniques. In addition, future in vivo studies are required to obtain knowledge about the long-term effects and the success rate of Endoflas with a follow-up period.

## Figures and Tables

**Table 1:** Descriptive statistics of dye leakage in several experimental groups.

Groups^*∗*^	Mean	±SD	±SE	Minimum	Maximum
GI	0.054	0.025	0.007	0.019	0.102
GII	0.133	0.091	0.026	0.044	0.277
GIII	0.146	0.066	0.019	0.047	0.230

^*∗*^Group I: endodontic pressure syringe; group II: modified disposable syringe; group III: reamer; SD, standard deviation; SE, standard error.

**Table 2:** Dunnett's T3 post hoc test.

Groups^*∗*^		Mean difference	*p*-value	95% CI
G1	GII	−0.07950	0.034^*∗∗*^	−0.15 to 0.0056
	GIII	−0.09283	0.001^*∗∗*^	−0.15 to 0.04
GII	GIII	−0.01333	0.966 NS	−0.097 to 0.07

^*∗*^Group I: endodontic pressure syringe; group II: modified disposable syringe; group III: reamer;  ^*∗∗*^Significant, *p* < 0.05; NS, not significant, *p* > 0.05. CI, confidence interval.

## Data Availability

The data used to support the findings of this study are included within the article.

## Abbreviations

ANOVA: Analysis of variance
Ca(OH)_2_: Calcium hydroxide
EDTA: Ethylenediaminetetraacetic acid
NaOCl: Sodium hypochlorite
ZOE: Zinc oxide eugenol.
